# The regrouping of Luminal B (HER2 negative), a better discriminator of outcome and recurrence score

**DOI:** 10.1002/cam4.5089

**Published:** 2022-07-31

**Authors:** Zheng‐Jun Yang, Yu‐Xiao Liu, Yue Huang, Zu‐Jin Chen, Hao‐Zhi Zhang, Yue Yu, Xin Wang, Xu‐Chen Cao

**Affiliations:** ^1^ The First Department of Breast Cancer Tianjin Medical University Cancer Institute and Hospital, National Clinical Research Center for Cancer Tianjin China; ^2^ Key Laboratory of Cancer Prevention and Therapy Tianjin China; ^3^ Key Laboratory of Breast Cancer Prevention and Therapy Tianjin Medical University, Ministry of Education Tianjin China; ^4^ Department of Thyroid and Neck Cancer Tianjin Medical University Cancer Institute and Hospital, National Clinical Research Center of Cancer, Tianjin's Clinical Research Center for Cancer Tianjin China

**Keywords:** breast cancer, breast cancer subtype, Luminal B, prognosis, recurrence score

## Abstract

**Background:**

Breast cancer (BC) remains the leading cause of cancer‐related deaths worldwide. High recurrence risk Luminal BC receives adjuvant chemotherapy in addition to standard hormone therapy. Considering the heterogeneity of Luminal B BC, a more accurate classification model is urgently needed.

**Methods:**

In this study, we retrospectively reviewed the data of 1603 patients who were diagnosed with HER2‐negative breast invasive ductal carcinoma. According to the expression level of PR and Ki‐67 index, the Luminal B (HER2‐negative) BCs were divided into three groups: ER+PR−Ki67_low_ (ER‐positive, PR‐negative, and Ki‐67 index <20%), ER+PR+Ki67_high_ (ER‐positive, PR‐positive, and Ki‐67 index ≥20%), and ER+PR−Ki67_high_ (ER‐positive, PR‐negative, and Ki‐67 index ≥20%). The cox proportional hazards regression model was used to evaluate the correlation between each variable and outcomes. Besides, discriminatory accuracy of the models was compared using the area under the receiver operating characteristic curve and log‐rank *χ* ^2^ value.

**Results:**

The analysis results showed that there was a significant correlation between subtypes using this newly defined classification and overall survival (*p* < 0.001) and disease‐free survival (DFS) (*p* < 0.001). Interestingly, patients in the ER+PR−Ki67_high_ subgroup have the worst survival outcome in Luminal B (HER2‐negative) subtype, similar to Triple‐negative patients. Besides, the ER+PR+Ki67_high_ has worse 5‐year DFS compared with Luminal A group. There was a significant relationship between the regrouping subtype and the recurrence score index (RI) (*p* < 0.001). Moreover, the results showed that patients in ER+PR–Ki67_high_ subtype were more likely to have high RI for distance recurrence (RI‐DR) and local recurrence (RI‐LRR). Our newly defined classification has a better discrimination ability to predict survival outcome and recurrence score of Luminal B (HER2‐negative) BC patients, which may help in clinical decision‐making for individual treatment.

## Graphical abstract

Our newly defined classification has a better discrimination ability to predict survival outcome and recurrence score of Luminal B (HER2 negative) breast cancer, which may help in clinical decision‐making for individual treatment.

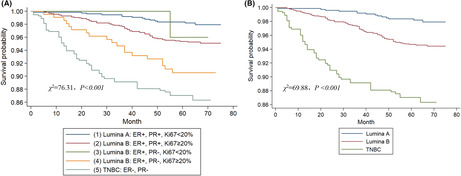

## 1 INTRODUCTION

According to the recently published data, breast cancer (BC) has become the world's most morbidity cancer and remains to be the major cause of cancer‐related deaths.^1^, ^2^ This scenario is plausibly due to the incomplete description of the biological heterogeneity of BC. Since 2011, molecular subtypes based on immunohistochemistry (IHC) have been considered one of the most valuable biomarkers to predict BC survival and treatment benefit. Although the multi‐gene expression molecular assays have superior accuracy and reproducibility, these assays are not available for all patients due to socioeconomic factors, especially in our developing countries. Thus, in our country, BC subtypes are still mainly determined using IHC surrogates.

Our previous study has revealed that Luminal B (HER2‐negative) type has a worse survival outcome at 10 years compared with Luminal A disease.^3^ What is more, nearly 20%–30% of patients with Luminal B BC may experience the risk of local or distant recurrence at 25 years from diagnosis. Thus, we hypothesize that risk stratification of BC patients with traditional molecular subtypes may not be accurate enough.

PR is an ER‐induced gene target and a modulator of ER behavior. However, the role of PR in BC etiology is still uncertain and whether its value can be used as a predictor in BC remains controversial. Furthermore, it is noteworthy that in the Luminal B subtype, both PR expression level and Ki‐67 index have a considerable range. Therefore, we assume that the Luminal B subtype is a more biological and clinically heterogeneous group that requires detailed classification to make individual treatment options.

The application of adjuvant chemotherapy in BC patients is generally based on molecular subtypes and other clinicopathological features (e.g., nodal status, histological grade). More recently, genomic tests such as the Oncotype Dx recurrence, Mammaprint, and EndoPredict have been used to guide the application of adjuvant chemotherapy. However, these tests have been developed mainly based on European and American populations and whether these tests are appropriate for Asian populations needs further validation.^4^, ^5^ RecurIndex is a multigene prognostic test developed mainly based on Asian populations. A recently published study showed that RecurIndex test might be more appropriate for Chinese patients than the Oncotype Dx in predicting recurrence.^6^, ^7^ Although RecurIndex is useful in our clinical treatment to predict the risk of recurrence and support treatment planning, this test has disadvantages such as high costs and long result cycles.

The purpose of this study is to investigate whether luminal B cancer can be divided into subgroups by PR values and Ki‐67 index to precisely predict clinical outcomes and support treatment planning. We also analyzed the association between recurrence score (RS) and our newly defined subgroups to investigate whether some patients may avoid the RS test before the starting of possible adjuvant chemotherapy due to high costs and a long time to get results.

## 2 PATIENTS AND METHODS

### 2.1 Patients

We conducted a retrospective analysis of 1603 patients diagnosed with ER+HER2− early stage invasive BC who underwent surgery in the Tianjin Medical University Cancer Institute and Hospital from August 2014 to December 2015. All patients received surgery. None of them received neoadjuvant therapy or had a prior history of cancer or bilateral tumors. Informed consent was obtained from all the patients above, and the Ethics Committees approved the research protocol for this study at the Tianjin Medical University Cancer Institute and Hospital.

Tumors were classified into four different subtypes according to the 2013 St. Gallen Consensus.^8^ When HER2 is positive, the patient is excluded.

### 2.2 Statistical analysis

Cox proportional hazards regression model (CPHRM) was used to analyze the association between each variable and study outcomes, including overall survival (OS) and disease‐free survival (DFS). These variables with *p* < 0.05 derived from univariate CPHRM were further carried out to perform forward stepwise regression. As a result, there were two variables screened out, including the T stage and N stage. We then built up a multivariate CPHRM adjusted for the T and N stages to evaluate the association between molecular subtypes and outcomes. Additionally, we predicted the 3‐ and 5‐year survival rates using the multivariate CPHRM, and the time‐dependent ROC curve was generated using the nearest neighbor method.^9^ The significance level was set to be 0.05. All analyses were performed using R software version 4.04 (The R Foundation for Statistical Computing). For all patients, follow‐up started from the date of operation. They were followed up in the manner we previously described.^3^ The last follow‐up was until December 30, 2020 and DFS.

## 3 RESULTS

### 3.1 Clinicopathological features and treatment modalities

A total of 1603 patients with early stage invasive BC were included. Clinicopathological features and treatment modalities are summarized in Table 1. The mean age of patients was 52 (range 22–74) years old. After surgery, 1447 patients received adjuvant chemotherapy and 447 patients received adjuvant radiotherapy. All patients with ER‐positive tumors received hormonal therapy. Most of the postmenopausal ER‐positive patients were treated with an aromatase inhibitor (AI). According to the STEPP analysis, all premenopausal patients with high risk were treated with ovarian function suppression (OFS) and AI.^10^ Our previous study showed that Luminal B patients have a higher frequency of lymph node metastasis than Triple‐negative patients.^3^ However, as shown in Table 1, patients with ER+PR−Ki67_high_ disease are less likely to have lymph node metastasis, compared with the other two subgroups (*p* < 0.001). Moreover, in the ER+PR−Ki67_high_ subgroup, patients are more likely to be histologic Grade III than the other two subgroups (*p* < 0.001). These results showed that patients with PR‐negative and high Ki‐67 index represent tumor biology more similar to that seen in Triple‐negative patients.

**TABLE 1:** Clinical–pathological features and treatment modalities at the presentation by breast cancer subtypes

Variables	Lumina A ER+, PR+ Ki67 <20% (*n* = 432)	Lumina B ER+, PR+ Ki67 ≥20% (*n* = 721)	Lumina B ER+, PR− Ki67 <20% (*n* = 45)	Lumina B ER+, PR− Ki67 ≥20% (*n* = 137)	TNBC ER−, PR− (*n* = 268)	Total (*n* = 1603)	*χ* ^2^ value	*p* value
Menopausal status								
No	207 (47.92%)	381 (52.84%)	9 (20.00%)	48 (35.04%)	125 (46.64%)	770 (48.03%)	*χ* ^2^ = 30.33	**<0.001**
Yes	225 (52.08%)	340 (47.16%)	36 (80.00%)	89 (64.96%)	143 (53.36%)	833 (51.97%)		
Chemotherapy								
No	72 (16.67%)	56 (7.77%)	5 (11.11%)	11 (8.03%)	12 (4.48%)	156 (9.73%)	*χ* ^2^ = 35.79	**<0.001**
Yes	360 (83.33%)	665 (92.23%)	40 (88.89%)	126 (91.97%)	256 (95.52%)	1447 (90.27%)		
Radiotherapy								
No	327 (75.69%)	497 (68.93%)	36 (80.00%)	102 (74.45%)	194 (72.39%)	1156 (72.11%)	*χ* ^2^ = 8.16	0.0859
Yes	105 (24.31%)	224 (31.07%)	9 (20.00%)	35 (25.55%)	74 (27.61%)	447 (27.89%)		
Endocrine therapy								
No	39 (9.03%)	40 (5.55%)	9 (20.00%)	28 (20.44%)	268 (100.00%)	384 (23.96%)	*χ* ^2^ = 1039.02	**<0.001**
Yes	393 (90.97%)	681 (94.45%)	36 (80.00%)	109 (79.56%)	0 (0.00%)	1219 (76.04%)		
Surgery								
MRM	386 (89.35%)	636 (88.21%)	41 (91.11%)	124 (90.51%)	239 (89.18%)	1426 (88.96%)	*χ* ^2^ = 1.04	0.9037
BCS	46 (10.65%)	85 (11.79%)	4 (8.89%)	13 (9.49%)	29 (10.82%)	177 (11.04%)		
Histologic grade								
I	32 (8.99%)	30 (4.62%)	1 (2.70%)	2 (1.71%)	1 (0.41%)	66 (4.70%)	*χ* ^2^ = 513.78	**<0.001**
II	322 (90.45%)	573 (88.29%)	36 (97.30%)	80 (68.38%)	91 (36.99%)	1102 (78.43%)		
III	2 (0.56%)	46 (7.09%)	0 (0.00%)	35 (29.91%)	154 (62.60%)	237 (16.87%)		
Lymphatic invasion								
No	380 (87.96%)	602 (83.50%)	43 (95.56%)	126 (91.97%)	247 (92.16%)	1398 (87.21%)	*χ* ^2^ = 19.73	**<0.001**
Yes	52 (12.04%)	119 (16.50%)	2 (4.44%)	11 (8.03%)	21 (7.84%)	205 (12.79%)		
T Stage								
T1	270 (62.50%)	394 (54.64%)	28 (62.22%)	63 (45.99%)	137 (51.12%)	892 (55.64%)		
T2	153 (35.42%)	307 (42.58%)	17 (37.78%)	71 (51.82%)	122 (45.52%)	670 (41.80%)	*χ* ^2^ = 21.47	**<0.001**
T3	9 (2.08%)	18 (2.50%)	0 (0.00%)	3 (2.19%)	8 (2.99%)	38 (2.37%)		
N Stage								
N0	293 (67.82%)	429 (59.50%)	27 (60.00%)	92 (67.15%)	182 (67.91%)	1023 (63.82%)	*χ* ^2^ = 11.	**0.0232**
N1	106 (24.54%)	207 (28.71%)	14 (31.11%)	29 (21.17%)	55 (20.52%)	411 (25.64%)		
N2	24 (5.56%)	51 (7.07%)	2 (4.44%)	12 (8.76%)	16 (5.97%)	105 (6.55%)		
N3	9 (2.08%)	34 (4.72%)	2 (4.44%)	4 (2.92%)	15 (5.60%)	64 (3.99%)		

Bold values indicate statistical significance was set as *p* < 0.05. Abbreviations: BCS, breast conserved surgery; MRM, modified radical mastectomy.

### 3.2 Outcomes, including recurrence, and survival

**FIGURE 1:**
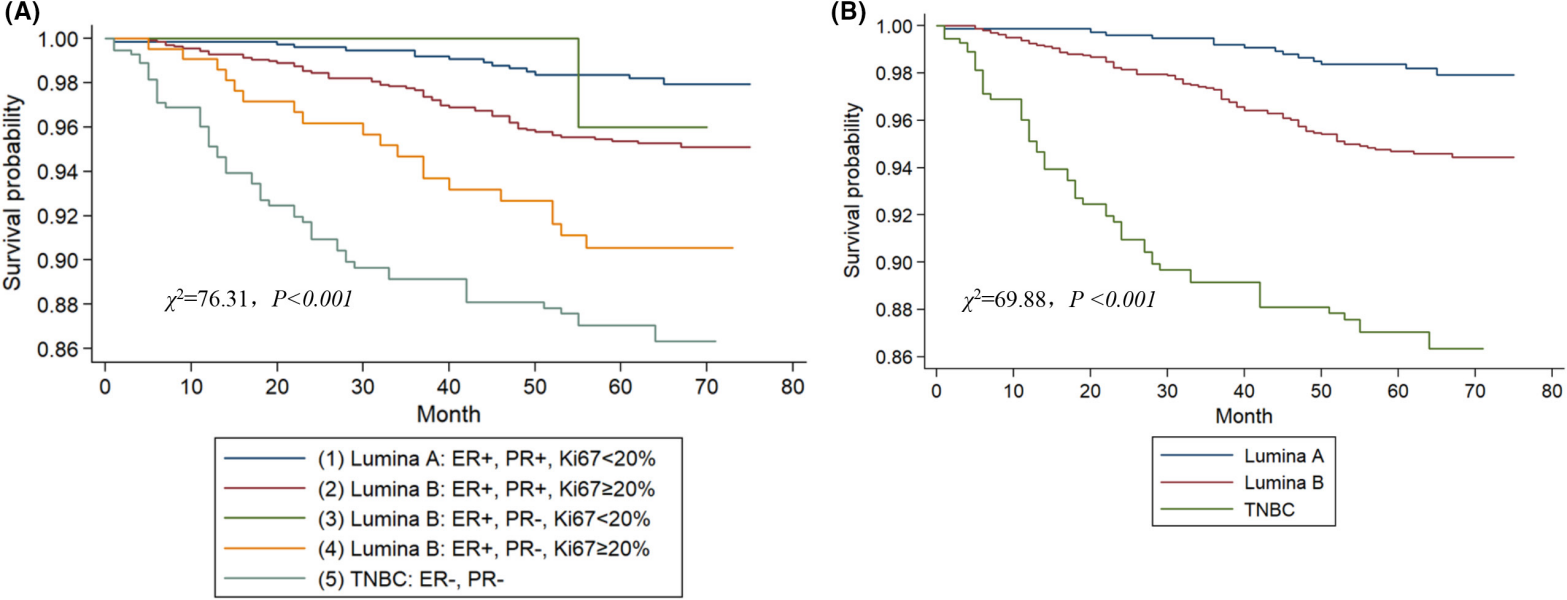
Adjusted survival analysis of the disease‐free survival according to the regrouping (A) and traditional Luminal B (HER2‐negative) breast cancer subtypes (B).

At the last time of follow‐up, 1448 (90.3%) patients were alive and disease free, 103 (6.4%) were alive with recurrent cancer, and 52 (3.3%) died of recurrent cancer. As shown in Tables 2 and 3; Tables S1 and S2, N stage (*p* < 0.001) and T stage (*p* < 0.001) were significantly associated with DFS and OS both in univariate and multivariate survival analyses. We then built up a multivariate CPHRM adjusted for the T stage and N stage to evaluate the association between molecular subtype and outcomes (Table S3). Kaplan–Meier survival curves comparing regrouping subtypes and 5‐year OS and 5‐year DFS were plotted. As shown in Figure 1, both regrouping and traditional subtypes significantly correlate with DFS in these patients (*p* < 0.001). The log‐rank *χ* ^2^ value of the regrouping model was higher than that of the traditional model in the adjusted survival analysis. As shown in Table 4, the differences between the new classifications are apparent, and each group is related to the prognosis. Interestingly, patients in the ER+PR−Ki67_high_ subgroup have the worst survival outcome in Luminal B (HER2‐negative) subtype, similar to Triple‐negative patients. Besides, the ER+PR+Ki67_high_ has worse 5‐year DFS compared with the Luminal A group. There was no significant difference in terms of 5‐year DFS between Luminal A and ER+PR−Ki67_low_ subgroup. Likewise, Kaplan–Meier survival curves compared the regrouping subtype and 5‐year OS in Figure S1. Taken together, both the regrouping and the traditional Luminal B (HER2‐negative) models were significant in the DFS of BC patients.

**TABLE 2:** Univariate analysis of clinicopathological variables affecting DFS

Variables	HR	95% CI	*Z* value	*p* value
Chemotherapy (yes vs. no)	1.2991	0.7236–2.3323	0.88	0.381
Radiotherapy (yes vs. no)	2.3053	1.6798–3.1638	5.17	**<0.001**
Menopausal status (yes vs. no)	0.8804	0.6418–1.2078	−0.79	0.430
Endocrine therapy (yes vs. no)	0.3652	0.2654–0.5027	−6.18	**<0.001**
Surgery (BCS vs. MRM)	0.7784	0.4535–1.3358	−0.91	0.363
Histologic Grade (III vs. I–II)	1.5131	0.9986–2.2926	1.95	0.051
Lymphatic invasion (yes vs. no)	1.2890	0.9480–1.7527	1.62	0.105
T stage (T3 vs. T1–2)	2.4293	1.8929–3.1178	6.97	**<0.001**
N stage (N2–3 vs. N0–1)	2.1380	1.8148–2.5188	9.09	**<0.001**

Bold values indicate statistical significance was set as *p* < 0.05. Abbreviations: BCS, breast conserved surgery; CI, confidence interval; DFS, disease‐free survival; HR, hazard ration; MRM, modified radical mastectomy.

**TABLE 3:** Multivariate analysis of clinicopathological variables affecting DFS

Variables	HR	95% CI	*Z* value	*p* value
T stage (T3 vs. T1–2)	1.7516	1.3368–2.2951	4.07	**<0.001**
N stage (N2–3 vs. N0–1)	1.8833	1.5737–2.2540	6.91	**<0.001**

Bold values indicate statistical significance was set as *p* < 0.05. Abbreviations: CI, confidence interval; DFS, disease‐free survival; HR, hazard ratio.

**TABLE 4:** Multivariate cox hazards regression model adjusted for T stage and N stage to evaluate the association between molecular subtype and DFS

Variables	Univariate cox hazards regression model				Multivariate cox hazards regression model			
	HR	95% CI	*Z* value	*p* value	HR	95% CI	*Z* value	*p* value
(1) Lumina A: ER+, PR+, Ki67 <20%	Ref				Ref			
(2) Lumina B: ER+, PR+, Ki67 ≥20%	2.7024	1.5131–4.8265	3.36	7.80E‐04	2.2237	1.2428–3.9787	2.69	**<0.001**
(3) Lumina B: ER+, PR−, Ki67 <20%	0.6933	0.0912–5.2724	−0.35	7.23E‐01	0.5967	0.0784–4.5413	−0.50	0.618
(4) Lumina B: ER+, PR−, Ki67 ≥20%	4.5879	2.3001–9.1515	4.32	1.53E‐05	4.1839	2.0960–8.3516	4.06	**<0.001**
(5) TNBC: ER−, PR−	7.5697	4.2180–13.5848	6.78	1.17E‐11	7.2778	4.0472–13.0873	6.63	**<0.001**
(2) Lumina B: ER+, PR+, Ki67 ≥20%	Ref				Ref			
(3) Lumina B: ER+, PR−, Ki67 <20%	0.2565	0.0356–1.8502	−1.35	1.77E‐01	0.2683	0.0372–1.9360	−1.30	0.192
(4) Lumina B: ER+, PR−, Ki67 ≥20%	1.6977	1.0152–2.8390	2.02	4.36E‐02	1.8815	1.1230–3.1524	2.40	**0.016**
(5) TNBC: ER−, PR−	2.8011	1.9543–4.0146	5.61	2.04E‐08	3.2729	2.2735–4.7114	6.38	**<0.001**
(3) Lumina B: ER+, PR−, Ki67 <20%	Ref				Ref			
(4) Lumina B: ER+, PR−, Ki67 ≥20%	6.6177	2.8859–49.4337	2.14	3.55E‐02	7.0116	2.9375–52.4393	2.10	**0.038**
(5) TNBC: ER−, PR−	10.9186	4.5119–78.8522	2.37	1.78E‐02	12.1964	4.6859–88.2354	2.48	**0.013**
(4) Lumina B: ER+, PR−, Ki67 ≥20%	Ref				Ref			
(5) TNBC: ER−, PR−	1.6499	1.2817–2.7731	2.08	3.87E‐02	1.7395	1.2346–2.9246	2.19	**0.037**

Bold values indicate statistical significance was set as *p* < 0.05. Abbreviations: CI, confidence interval; DFS, disease‐free survival; HR, hazard ratio.

### 3.3 Discrimination of models

**FIGURE 2:**
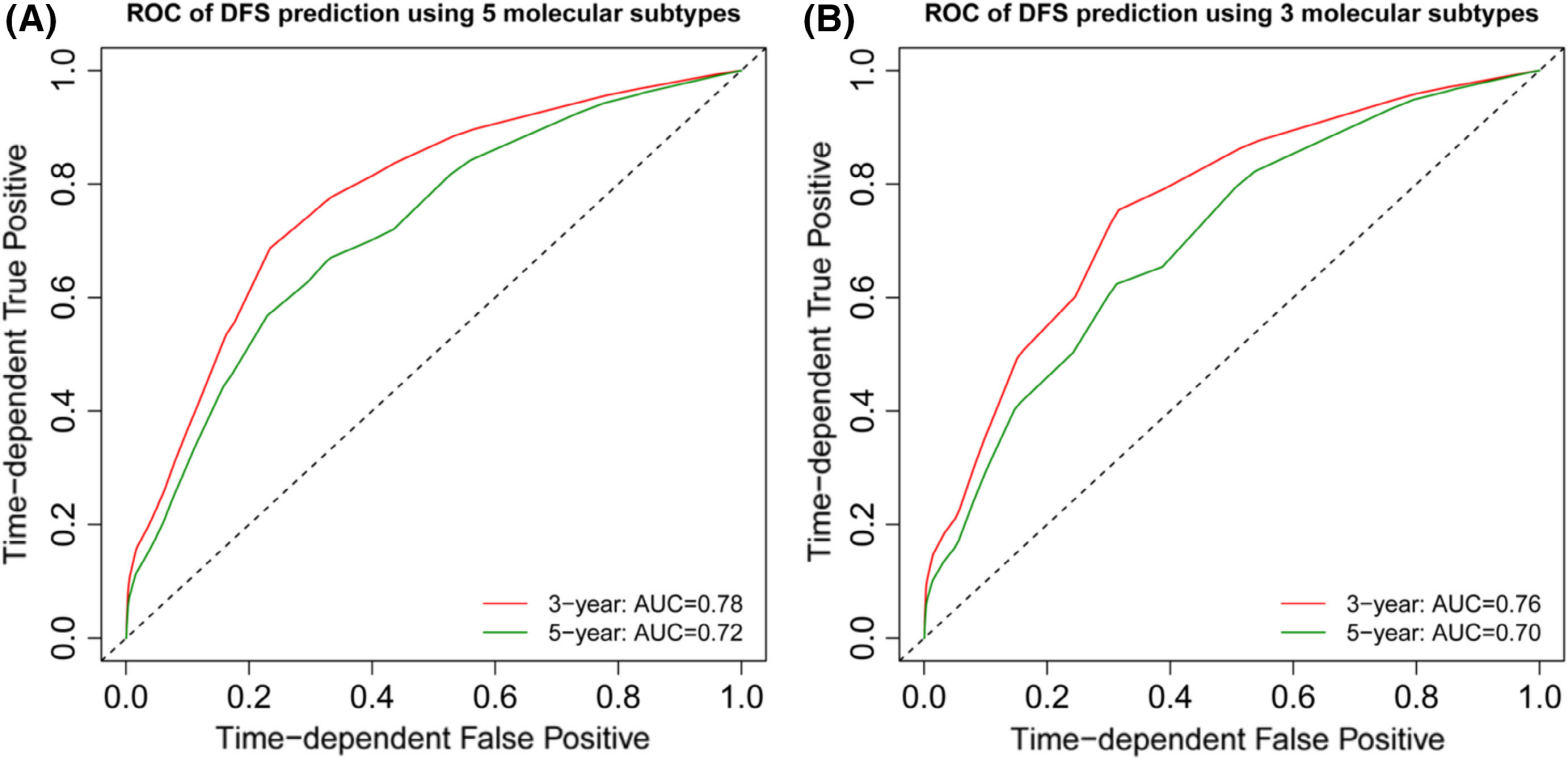
Receiver operating characteristic curve for predictability of survival outcome in breast cancer patients according to the regrouping (A), and traditional Luminal B (HER2‐negative) breast cancer subtypes (B).

To further validate the predictive value of the regrouping model, the nearest neighbor method was used to construct a time‐dependent ROC curve. As shown in Figure 2, the area under the receiver operating characteristic curve (AUROC) for 3‐ and 5‐year DFS were 0.78, 0.72 and 0.76, 0.70 for regrouping and traditional sets, respectively. Thus, our result suggested that the regrouping model had a better discrimination ability compared with the traditional model. Besides, as shown in Figure S2, the regrouping model also had well discrimination for OS. Collectively, these results indicated a good performance of the regrouping model for both DFS and OS prediction.

### 3.4 Associations between subtypes and recurrence index category

As shown in Tables 5 and 6, univariate analyses revealed a significant relationship between subtype and recurrence index both for distance recurrence (RI‐DR) and local recurrence (RI‐LRR) (*p* < 0.001). And the results showed that the Luminal B BC patients with ER+PR−Ki67_high_ tumors were more likely to have a high risk both for RI‐DR and RI‐LRR: 18 of 27 (66.67%) patients had a high risk for RI‐DR and 16 of 27 (59.26%) patients had a high risk for RI‐LRR.

**TABLE 5:** Associations between clinical and pathological features and recurrence index for distance recurrence

Characteristic	Total	Stratification by RI‐DR		*p* value
	*n* = 178	High risk, *n* = 47	Low risk, *n* = 131	
Subtype				**<0.001**
ER+, PR+, Ki67 >20	52 (27.23%)	15 (31.91%)	37 (28.24%)	
ER+, PR+, Ki67 ≤20	80 (41.88%)	10 (21.28%)	70 (53.44%)	
ER+, PR−, Ki67 >20	27 (14.14%)	18 (38.30%)	9 (6.87%)	
ER+, PR−, Ki67 ≤20	19 (9.95%)	4 (8.51%)	15 (11.45%)	
Age				0.2
<40	22 (12.36%)	9 (19.15%)	13 (9.92%)	
40–60	101 (56.74%)	27 (57.45%)	74 (56.49%)	
>60	55 (30.90%)	11 (23.40%)	44 (33.59%)	
N stage				**<0.001**
0	152 (85.39%)	34 (51.06%)	128 (97.71%)	
1	26 (14.61%)	26 (48.94%)	3 (2.29%)	
Tumor stage				**≤0.001**
1	96 (53.93%)	14 (29.79%)	82 (62.60%)	
2	82 (46.07%)	33 (70.21%)	49 (37.40%)	
Grade				**<0.001**
I	7 (3.93%)	1 (2.13%)	6 (4.58%)	
II	151 (84.83%)	27 (57.45%)	124 (94.66%)	
III	20 (11.24%)	19 (40.43%)	1 (0.76%)	
LVI				**<0.001**
Yes	67 (37.64%)	37 (78.72%)	30 (22.90%)	
No	111 (62.36%)	10 (21.28%)	101 (77.10%)	

Bold values indicate statistical significance was set as *p* < 0.05. Abbreviations: LVI, lymphovascular invasion; RI‐DR, recurrence index for distance recurrence.

**TABLE 6:** Associations between clinical and pathological features and recurrence index for local recurrence

Characteristic	Total	Stratification by RI‐LRR		*p* value
	*n* = 178	High risk, *n* = 49	Low risk, *n* = 129	
Subtype				**<0.001**
ER+, PR+, Ki67 >20	52 (29.21%)	18 (36.73%)	34 (26.36%)	
ER+, PR+, Ki67 ≤20	80 (44.94%)	9 (18.37%)	71 (55.04%)	
ER+, PR−, Ki67 >20	27 (15.17%)	16 (32.65%)	11 (8.53%)	
ER+, PR−, Ki67 ≤20	19 (10.67%)	6 (12.24%)	13 (10.08%)	
Age				**0.036**
<40	22 (12.36%)	11 (22.45%)	11 (8.53%)	
40–60	101 (56.74%)	26 (53.06%)	75 (58.14%)	
>60	55 (30.90%)	12 (24.49%)	43 (33.33%)	
N stage				**<0.001**
0	152 (85.39%)	28 (57.14%)	124 (96.12%)	
1	26 (14.61%)	21 (42.86%)	5 (3.88%)	
Tumor stage				0.2
1	96 (53.93%)	23 (46.94%)	73 (56.59%)	
2	82 (46.07%)	26 (53.06%)	56 (43.41%)	
Grade				**<0.001**
I	7 (3.93%)	1 (2.04%)	6 (4.65%)	
II	151 (84.83%)	34 (69.39%)	117 (90.70%)	
III	20 (11.24%)	14 (28.57%)	6 (4.65%)	
LVI				**<0.001**
Yes	67 (37.64%)	38 (77.55%)	29 (22.48%)	
No	111 (62.36%)	11 (22.45%)	100 (77.52%)	

Bold values indicate statistical significance was set as *p* < 0.05. Abbreviations: LVI, lymphovascular invasion; RI‐LRR, recurrence index for local recurrence.

## 4 DISCUSSION

BC can be divided into different subtypes based on gene expression profiling, and each subtype represents a distinct clinical outcome.^11^, ^12^, ^13^ Considering the heterogeneity of Luminal B cancer, a more accurate classification model is urgently needed. It is noteworthy that both PR expression level and Ki‐67 index have a considerable range in the Luminal B subtype. To analyze whether the PR expression level plays an essential role in affecting the survival outcome of Luminal B (HER2‐negative) BC patients. In this present study, according to the expression level of PR and Ki‐67 index, the Luminal B (HER2‐negative) BCs were divided into three subgroups.

The results showed that patients with PR‐negative have worse survival outcomes compared with PR‐positive in high ki‐67 index Luminal B (HER2‐negative) BC (as shown in Table 2). However, when the ki‐67 index is low, there was no significant difference between PR‐negative and PR‐positive (Luminal A) patients. Our present results agree with recently published studies showing a stronger prognostic value of PR status in high proliferating tumors.^14^, ^15^ So, we should consider both PR expression and the Ki‐67 index to make accurate predictions about the survival outcome of Luminal B (HER2‐negative) patients. Thus, the Luminal B (HER2‐negative) BCs were newly divided into three subgroups for further risk stratification. In our present study, the results showed a significant difference among the three subgroups in terms of survival outcomes while patients with ER+PR−Ki67_high_ tumors had the worst survival among Luminal B (HER2‐negative) patients.

To assess whether the regrouping model has better discrimination ability, AUROC was calculated. The results showed that the regrouping model had better discrimination with a higher AUROC value for prediction of the 3‐year DFS, 5‐year DFS, respectively. Besides, the regrouping model showed a greater log‐rank *χ* ^2^ value than the traditional model, indicating this new classification could better predict the prognosis of Luminal B patients than the traditional classification. Based on these results, we supposed that these three subgroups should be treated separately.

Our present study showed that PR status was related to poor survival outcomes when Ki67 status was high. The recently published paper found that PR low‐expression tumors had significantly higher median SUVs than PR high‐expression tumors, suggesting that low‐PR expression can identify a high RS in ER+HER2− disease.^16^, ^17^

Endocrine therapy is still the cornerstone for primary Luminal B (HER2‐negative) BC treatment. However, a significant proportion of patients may experience endocrine resistance from the beginning or during the treatment process.^18^, ^19^ Although the updated results from SOFT and TEXT showed that premenopausal women with high‐risk hormone receptor‐positive HER2‐negative tumors can achieve survival improvements significantly through the combination with OFS.^20^ As to PR low or negative expression patients, whether they can achieve survival improvements from the addition of OFS is still controversial. Based on published work, we can deduce that patients in the PR low or negative group may resist antihormonal therapies.^21^, ^22^ So, we suggested that patients with ER‐positive PR‐negative tumors can be treated similarly to Triple‐negative cancer patients with intensive chemotherapy.

RNA profiling has determined that ER+PR− tumors represent a subgroup of BC with poor survival outcomes and high invasive features, although being clinically ER+ by IHC test.^23^ Consist with this result, our present study also showed that the ER+PR−Ki67_high_ group usually had a higher risk of grade III and worse survival outcomes similar to the Triple‐negative group. However, not all ER+PR− groups had poor survival outcomes. As shown in Figure S1, the ER+PR−Ki67_low_ group had similar survival outcomes to the Luminal A group, a subset recognized for its indolent behavior. This result was consistent with the published data, which showed that some ER+PR− tumors had ER+PR+ gene expression profiles. A proportion of ER+PR− tumors showed a pattern of ER−PR− gene expression. Similar to our findings, a recently published study showed that 16% of IHC ER+HER2− BCs could be reclassified as Basal molecular subtypes by multigene expression profiling.^24^ Furthermore, studies showed that the ER+HER2− Basal subtype is obviously different from the ER+HER2− Luminal subtype and sometimes similar to the ER‐negative Basal subtype with regard to potential therapeutic sensitivity to systemic therapies.^24^, ^25^, ^26^

Currently, multigene expression profiles, such as Oncotype DX, MammaPrint, and Prosiga, have become widely used to predict the risk of recurrence and adjuvant chemotherapy benefits.^4^, ^27^, ^28^, ^29^ However, these genomic‐based classification applications are restricted in our developing countries because of the relatively higher price. On the other hand, the indications for these genetic tests are too broad, easily cause abuse, and bring unnecessary economic burdens. The surrogate histopathological definitions we used in this present study are widely affordable and using these histopathological‐based regrouping can discriminate low‐risk or high‐risk tumors in Luminal B HER2‐negative BC. Our study shows that a high Ki‐67 index and PR‐negative status are potential predictive factors for high RS both for distance recurrence and local recurrence in Luminal B HER2‐negative BC patients.

Moreover, the purpose of the MINDACT study is to select patients from high clinical relapse risk using a 70‐gene signature to avoid unnecessary adjuvant chemotherapy. Nevertheless, this study did not include the expression of PR and Ki‐67, which leads to an incomplete definition of high‐risk patients. Our study used the expression levels of PR and Ki67 to supplement the definition of high‐risk groups. More importantly, discordances in estimates occur between the genomic tests due to their different emphasis on ER‐related features and proliferation features,^30^ which reflect the importance of a combined analysis with clinical–pathological features. Recently, several studies aimed to integrate the 21‐Gene RS and clinical–pathological features to individualize prognosis and prediction of chemotherapy benefit.^31^, ^32^, ^33^ However, none of them use the expression of PR and Ki67. In addition, some studies using DNA methylation data have also revealed the vast heterogeneity of epigenetic levels between luminal breast tumors and constructed molecular classifications.^34^, ^35^, ^36^, ^37^ However, these findings seem to be far from being clinically used. So, Ki‐67 index and PR status should be considered before using gene expression profiles, which would allow some patients with ER+PR−Ki67_high_ tumors to omit the gene expression profiling tests before receiving adjuvant chemotherapy. So, to some extent, the regrouping model can replace the expensive genomic assay in Luminal B patients.

Our study has several strengths. First, according to the PR status and Ki‐67 index, the Luminal B (HER2‐negative) BC were divided into three subgroups. And we have proved that this new classification could better predict the prognosis of Luminal B patients than the traditional classification. Our research optimizes the traditional molecular subtype and shows that a high Ki‐67 index and PR‐negative status are potential predictive factors for high RS in Luminal B HER2‐negative BC patients. PR‐negative and high Ki‐67 index may represent tumor biology more similar to that seen in Triple‐negative patients. So, Ki‐67 index and PR status may be considered before using gene expression profiles, which would allow some patients with high Ki‐67 index and PR‐negative status tumors to omit the gene expression profiling tests before receiving adjuvant chemotherapy. Therefore, reorganizing Luminal B (HER2‐negative) BC by measuring the expression of PR and Ki67 instead of gene expression has clinical significance for accurately assessing the prognosis of BC and providing a reliable basis for clinical treatment.

Some limitations should be taken into account when applying our results. First, we used a retrospective design with small sample size. Thus, the results should be considered exploratory and hypothetical. Second, due to a limited follow‐up period, the number of patients with ER+PR−Ki67_low_ BC is quite a few, with no death and only one local recurrence observed in our present study. Therefore, our findings should be validated in a large, multi‐center study.

## 5 CONCLUSION

The reclassification of Luminal B (HER2‐negative) BC using the expression of PR and Ki67 is of clinical significance. Patients with ER+PR−Ki67_high_ tumors differ from the other two groups in survival outcomes and treatment response, who cannot be continued treated as a homogeneous group. Besides, these patients were more likely to have high RS scores to receive adjuvant chemotherapy. Thus, we recommend that Luminal B BC patients with PR‐negative and high Ki67 index may avoid RS test and accept intensive adjuvant chemotherapy.

## AUTHOR CONTRIBUTIONS

Zheng‐Jun Yang: Investigation, data analysis and interpretation, methodology, visualization, writing—original draft, writing review, and editing. Yu‐Xiao Liu: Investigation, data analysis and interpretation, methodology, visualization, writing—original draft, writing review, and editing. Yue Huang: Investigation, data acquisition, interpretation, editing. Zu‐Jin Chen: Investigation, data acquisition, interpretation, editing. Hao‐Zhi Zhang: Investigation, data acquisition, interpretation, editing. Yue Yu: Investigation, data acquisition, interpretation, editing, and supervision. Xin Wang: Investigation, data acquisition, interpretation, editing, and supervision. Xu‐Chen Cao: Investigation, data acquisition, interpretation, editing, and supervision.

## FUNDING INFORMATION

This study was supported by the National Natural Science Foundation of China (grant nos. 81372843, 81472472, and 81502518).

## CONFLICT OF INTEREST

None.

## Supporting information


Figure S1

Figure S2

Table S1

Table S2

Table S3

## Data Availability

All data generated or analyzed during this study are included in this article and its supplementary information files.
