# Supplementary figures and images for: The regrouping of Luminal B (HER2 negative), a better discriminator of outcome and recurrence score
Source: Cancer Med. 2022 Jul 31;12(3):2493–504. doi: 10.1002/cam4.5089 (PMC9939104; doi:10.1002/cam4.5089)

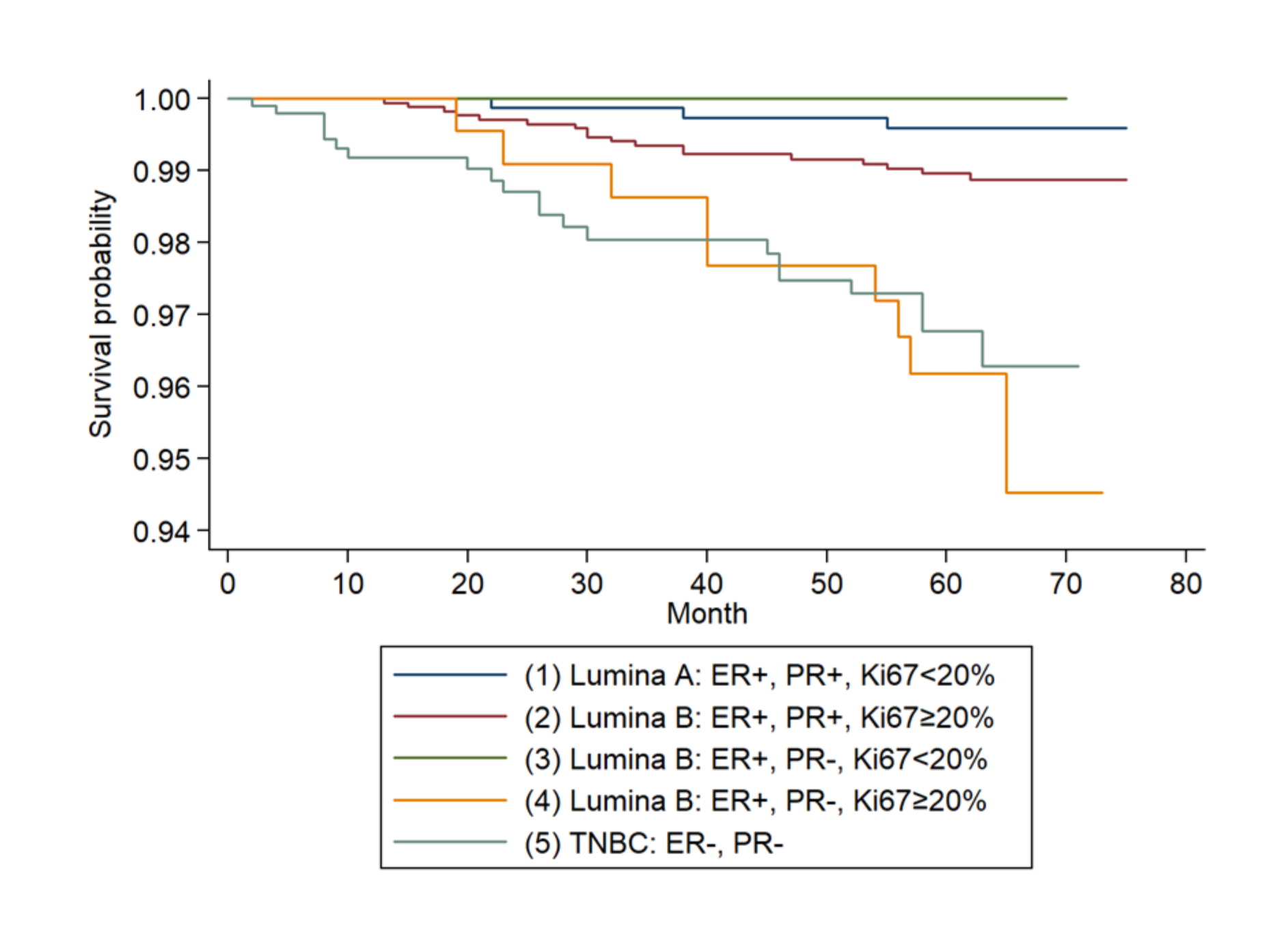

Supplement: Supplementary file 1 — Figure S1 [file CAM4-12-2493-s001.tif]

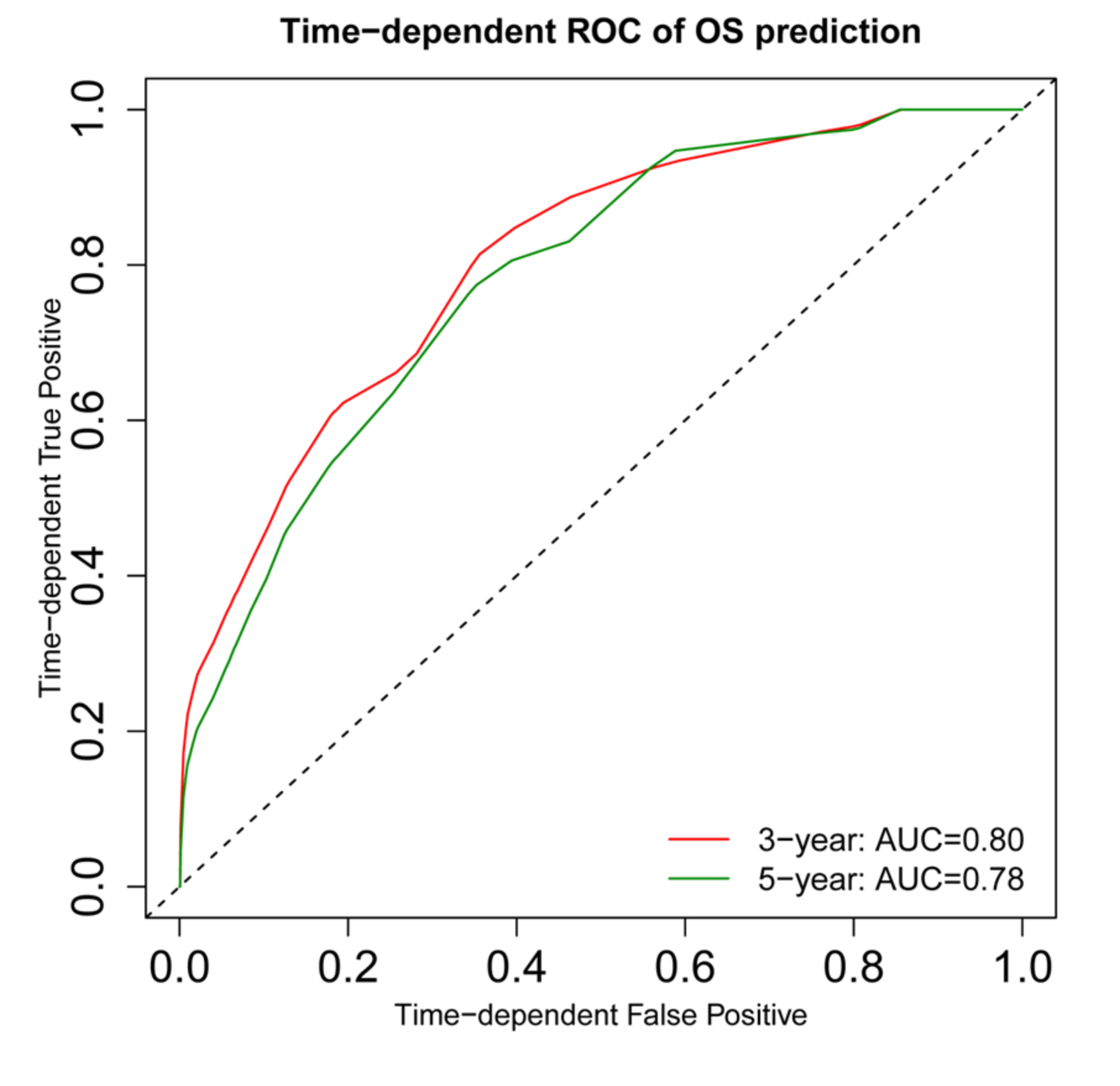

Supplement: Supplementary file 2 — Figure S2 [file CAM4-12-2493-s002.tif]
