# Supplementary material for: The regrouping of Luminal B (HER2 negative), a better discriminator of outcome and recurrence score
Source: Cancer Med. 2022 Jul 31;12(3):2493–504. doi: 10.1002/cam4.5089 (PMC9939104; doi:10.1002/cam4.5089)
Supplement: Supplementary file 3 — Table S1 [file CAM4-12-2493-s005.docx]

Table S1: Univariate analysis of clinicopathological variables affecting OS

| Variables | HR | 95% CI | | *Z* value | *P* value |
| --- | --- | --- | --- | --- | --- |
| Chemotherapy(Yes vs No) | 1.2735 | 0.4588 | 3.5348 | 0.46 | 6.43E-01 |
| Radiotherapy (Yes vs No) | 2.7615 | 1.5960 | 4.7781 | 3.63 | 2.82E-04 |
| Menopausal Status (Yes vs No) | 1.5671 | 0.8878 | 2.7664 | 1.55 | 1.21E-01 |
| Endocrine therapy (Yes vs No) | 0.3296 | 0.1899 | 0.5721 | -3.95 | 7.97E-05 |
| Surgery (BCS vs MRM) | 0.6661 | 0.2423 | 1.8308 | -0.79 | 4.31E-01 |
| Histologic Grade (III vs I-II) | 1.9946 | 0.9919 | 4.0108 | 1.94 | 5.27E-02 |
| Lymphatic invasion (Yes vs No) | 1.2161 | 0.7069 | 2.0920 | 0.71 | 4.80E-01 |
| T stage (T3 vs T1-2) | 2.9324 | 2.0249 | 4.2466 | 5.69 | 1.24E-08 |
| N stage (N2-3 vs N0-1) | 2.5290 | 1.9433 | 3.2914 | 6.90 | 5.12E-12 |

MRM: Modified radical mastectomy BCS: Breast conserved surgery
