# Supplementary material for: The regrouping of Luminal B (HER2 negative), a better discriminator of outcome and recurrence score
Source: Cancer Med. 2022 Jul 31;12(3):2493–504. doi: 10.1002/cam4.5089 (PMC9939104; doi:10.1002/cam4.5089)
Supplement: Supplementary file 4 — Table S2 [file CAM4-12-2493-s003.docx]

Table S2: Multivariate analysis of clinicopathological variables affecting OS

| Variables | HR | 95% CI | | *Z* value | *P* value |
| --- | --- | --- | --- | --- | --- |
| T stage (T3 vs T1-2) | 1.8846 | 1.2047 | 2.9483 | 2.78 | 5.51E-03 |
| N stage (N2-3 vs N0-1) | 2.1914 | 1.6764 | 2.8646 | 5.74 | 9.46E-09 |
