# Supplementary material for: The regrouping of Luminal B (HER2 negative), a better discriminator of outcome and recurrence score
Source: Cancer Med. 2022 Jul 31;12(3):2493–504. doi: 10.1002/cam4.5089 (PMC9939104; doi:10.1002/cam4.5089)
Supplement: Supplementary file 5 — Table S3 [file CAM4-12-2493-s004.docx]

Table S3 Multivariate Cox hazards regression model adjusted for T stage and N stage to evaluate the association between molecular subtype and OS

| Variables | Univariate Cox hazards regression model | | | | | Multivariate Cox hazards regression model | | | | |
| --- | --- | --- | --- | --- | --- | --- | --- | --- | --- | --- |
|  | HR | 95% CI | | *Z* value | *P* value | HR | 95% CI | | *z* value | *P* value |
| (1) Lumina A: ER+, PR+, Ki67<20% | Ref |  |  |  |  | Ref |  |  |  |  |
| (2) Lumina B: ER+, PR+, Ki67≥20% | 3.6347 | 1.0707 | 12.3394 | 2.07 | 3.85E-02 | 2.7934 | 0.8204 | 9.5113 | 1.64 | 1.00E-01 |
| (3) Lumina B: ER+, PR-, Ki67<20% | - | - | - | - | - | - | - | - | - | - |
| (4) Lumina B: ER+, PR-, Ki67≥20% | 9.9630 | 2.6963 | 36.8139 | 3.45 | 5.66E-04 | 8.4092 | 2.2734 | 31.1054 | 3.19 | 1.42E-03 |
| (5) TNBC: ER-, PR- | 12.7855 | 3.8121 | 42.8813 | 4.13 | 3.67E-05 | 12.5432 | 3.7222 | 42.2688 | 4.08 | 4.50E-05 |
| (2) Lumina B: ER+, PR+, Ki67≥20% | Ref |  |  |  |  | Ref |  |  |  |  |
| (3) Lumina B: ER+, PR-, Ki67<20% | - | - | - | - | - | - | - | - | - | - |
| (4) Lumina B: ER+, PR-, Ki67≥20% | 2.7411 | 1.2307 | 6.1053 | 2.47 | 1.36E-02 | 3.0104 | 1.3488 | 6.7187 | 2.69 | 7.13E-03 |
| (5) TNBC: ER-, PR- | 3.5176 | 1.8728 | 6.6071 | 3.91 | 9.20E-05 | 4.4903 | 2.3598 | 8.5443 | 4.58 | 4.75E-06 |
| (3) Lumina B: ER+, PR-, Ki67<20% | Ref |  |  |  |  | Ref |  |  |  |  |
| (4) Lumina B: ER+, PR-, Ki67≥20% | - | - | - | - | - | - | - | - | - | - |
| (5) TNBC: ER-, PR- | - | - | - | - | - | - | - | - | - | - |
| (4) Lumina B: ER+, PR-, Ki67≥20% | Ref |  |  |  |  | Ref |  |  |  |  |
| (5) TNBC: ER-, PR- | 1.2833 | 0.5877 | 2.8021 | 0.63 | 5.31E-01 | 1.4916 | 0.6808 | 3.2681 | 1.00 | 3.18E-01 |
